# Development of an assessment strategy in preclinical fixed prosthodontics course using virtual assessment software—Part 1

**DOI:** 10.1002/cre2.109

**Published:** 2018-05-31

**Authors:** Ramtin Sadid‐Zadeh, Dillon Feigenbaum

**Affiliations:** ^1^ Department of Restorative Dentistry University at Buffalo School of Dental Medicine New York USA

**Keywords:** compare software, dental education, fixed prosthodontics, virtual assessment

## Abstract

The purpose of this study was to develop an assessment strategy for preclinical foxed prosthodontics using a virtual assessment software. This descriptive study examined 80 collected ivory teeth from previous classes prepared during preclinical fixed prosthodontics course. Ivory teeth prepared for the complete cast (tooth no. 46), metal ceramic (teeth nos 24 and 46), and all ceramic (tooth no. 21) crowns were scanned and superimposed with their respective standard preparations. Differences between these teeth and standard teeth prepared by faculty were computed using Compare software to generate comparison percentages. In addition, average finish line width, average total occlusal convergence, average axial wall height, and undercut presence/absence were quantified for student preparations using the software. Software‐generated values were then descriptively compared with faculty assessments. Comparison percentages aligned with faculty assessments for the amount of occlusal/incisal reduction and finish line location. The average axial wall height and finish line width calculated by Compare software were grouped based on their respective faculty assessment criteria. Software‐generated comparison percentages may be used to assess the amount of occlusal/incisal reduction and finish line location in student preparations. Additionally, averages extracted from Compare software could be used to assess student performance for axial wall height and finish line width.

## 1 INTRODUCTION

Assessment of tooth prepared by students in preclinical environment is a critical component of dental education in fixed prosthodontics. It is imperative that students receive consistent feedback from dental faculty in order to continually improve their performance before advancing to clinical practice. Lack of consistency in assessment may lead to confusion in students' understanding of the principles of tooth preparation, as well as lack of improvement in the psychomotor skills required for tooth preparation.

In 1982, Mackenzie, Antonson, Weldy, and Simpson (1982) described 16 areas that contribute to inconsistent assessment, including checkpoint ambiguity, faculty memory, incomplete coverage of dimensions, unspecified exceptions, untrained estimation of size, undersized aids to judgment, unspecified methods of observing, incomplete operational definitions, unsystematic inspections, discrepancies in visual acuity, degrees of leniency, inadequacy of verbal definitions, inadequate communication with nonverbal examples, definition ambiguities, differences in background, and differences in mental processing. To address the many factors that contribute to lack of faculty agreement about student work, including grading scales, rater calibration, training, and subjective influences, Knight (1997) suggested implementation of faculty calibration and well‐defined grading criteria in order to overcome problems of inconsistent faculty assessment (Feil & Gatti, 1993). However, even after calibrating faculty evaluation of student work as acceptable or unacceptable, faculty members still frequently mark unacceptable student work as acceptable in practice (Haj‐Ali & Feil, 2006). This issue becomes even more difficult when calibrating faculty for the use of larger grading scales (Fuller, 1972; Haj‐Ali & Feil, 2006; Lilley, Bruggen Cate, Holloway, Holt, & Start, 1968; Salvendy, Hinton, Ferguson, & Cunningham, 1973; Sharaf, AbdelAziz, & El Meligy, 2007). Faculty assessment is also complicated by significant levels of disagreement that are often observed between graders when evaluating student performance (Fuller, 1972; Lilley et al., 1968; Salvendy et al., 1973). Moreover, when a grader evaluates the same work on separate occasions, discordance in grading has been observed (Fuller, 1972; Lilley et al., 1968; Salvendy et al., 1973). Despite all of the variables involved in assessment of student performance, the Commission on Dental Accreditation mandates use of assessment forms and faculty calibration for U.S. dental schools (American Dental Association, 2006).

In an attempt to eliminate human bias from assessment of dental student work, E4D Technologies developed a virtual assessment tool. In 2013, Renne et al. suggested that technology could provide an alternative to faculty grading of dental student performance. This study reported that software could be used to consistently and reliably compare student tooth preparations to standard preparations. They concluded that the numerical comparison generated by the software is more precise than faculty assessments. However, in 2015, Callan, Haywood, Cooper, Furness, and Looney found no correlation between faculty assessments and the percentage comparison values computed by the software. This discrepancy may arise from the fact that the percentage comparison feature does not assess numerous criteria considered fundamental to tooth preparation that are assessed by faculty. Therefore, the purpose of this descriptive study was to develop a quantitative assessment strategy to accompany the use of Compare software by considering the fundamental of tooth preparation for evaluating amount of occlusal/incisal reduction (O/IR), finish line location (FLL) and finish line width (FLW), axial wall height (AWH), total occlusal convergence (TOC), and presence or absence of undercut. The use of Compare software may provide an objective measurement when evaluating preparation for complete coverage restoration in preclinical setting.

## 2 METHODS

A preexisting assessment form for fixed prosthodontics from the University at Buffalo School of Dental Medicine (UB SDM) was used as a template for this study. Traditionally, the following criteria have been used to evaluate students' performance for preparation of complete coverage restorations, by calibrated faculty members at UB SDM: (1) adjacent teeth and soft tissue; (2) amount of O/IR; (3) TOC; (4) presence/absence of undercut; (5) contour and long axis of tooth preparation; (6) AWH; (7) FLW; (8) FLL; and (9) finish of the preparation. For the criteria stated above, Numbers 2, 3, 4, 6, 7, and 8 may be evaluated by Compare software (Planmeca/E4D Technologies, Richardson, TX, USA). Specifically, the Compare software may be used to evaluate student tooth preparations against standard tooth preparations based on the following criteria: (a) comparison percentage (comparison%); (b) FLW (termed shoulder width); (c) TOC; (d) AWH; and (e) presence/absence of undercut. Compare software is designed to calculate, the average values of FLW, TOC, and AWH criteria and the presence/absence of undercut for preparation of complete coverage restorations could be calculated independent to the standard preparations. The standard preparation is defined as teeth prepared by the faculty following the design and the amount of reduction presented to students. However, to determine comparison%, the software calculates the discrepancy in reduction (over‐reduction or under‐reduction) between student preparations and standard preparations. The software calculates the percentage of the surface area of the student preparation that fell within the tolerable range of discrepancy from the standard preparation. The area within tolerances is defined as the comparison%.

**Figure 1:**
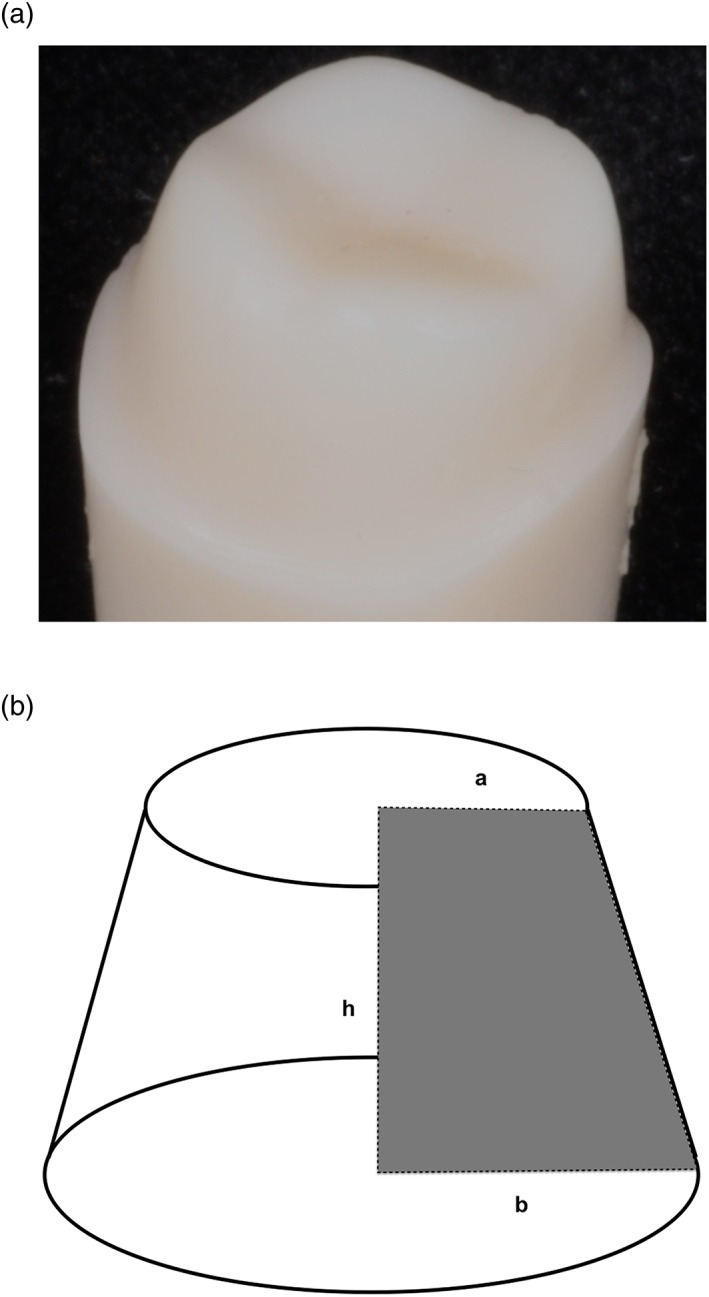
(a) Tooth prepared for a full coverage restoration. (b) Frustum of a cone

Teeth have a complicated morphology; as a result, it would be mathematically impossible to calculate the actual surface area for a tooth prepared for a complete coverage restoration. However, when the complex anatomy of a prepared tooth is simplified, it resembles a frustum of a cone (Figure 1). Then, the surface area of a tooth prepared for a complete coverage restoration can be estimated using the following formula:

$$\text{Area}\;=\;\mathrm{Top}\;\text{Circle Area}\;+\;\text{Lateral Area of Frustum of}\;a\;\text{Cone}\text{Area}\;=\;\pi \;a^{2}+\;\pi \;\left(a\;+\;b\right)\;\surd {\left(b-a\right)}^{2}+\;h^{2},$$

where “h” is the height of the tooth from finish line to occlusal table, “b” is the diameter of the tooth at the finish line, and “a” is the radius of the tooth on the occlusal table. The comparison% calculates the percentage of the surface area matching the standard preparation. On the basis of the formula above, the comparison% should be influenced by the amount of O/IR and the FLL.

**Figure 2:**
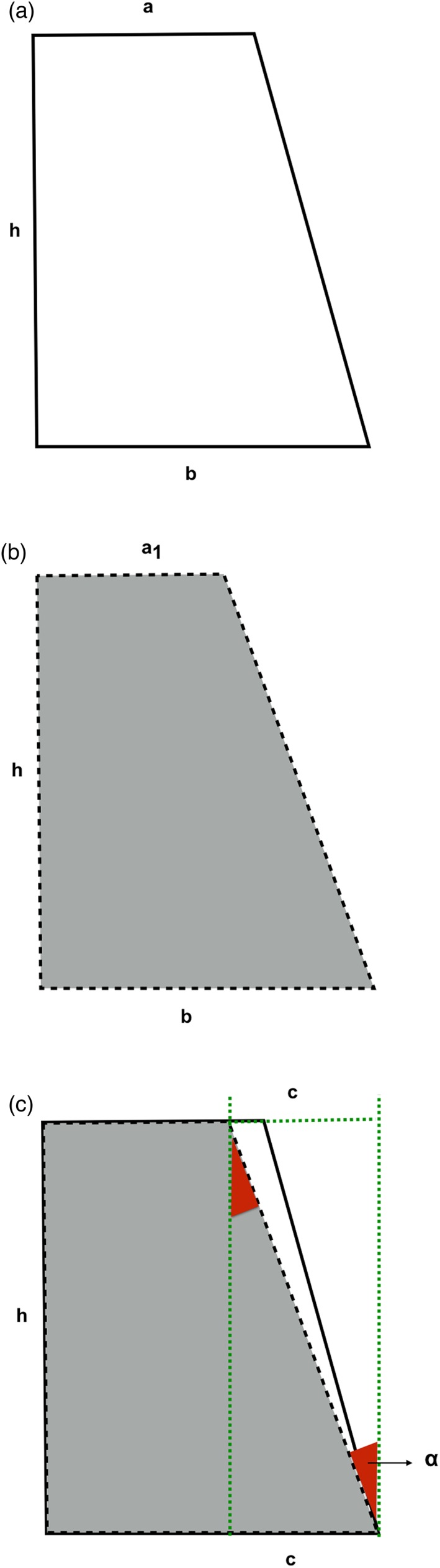
(a) Two‐dimensional view of tooth preparation with ideal total occlusal convergence. (b) Two‐dimensional view of tooth preparation with increased total occlusal convergence. (c) Superimposed of (a) and (b)

Figure 2 shows a two‐dimensional schematic of a frustum of a cone. In dentistry, with a constant height “h,” the radius “a” decreases to “a_1_” for the same tooth preparation with increased TOC. It is therefore possible to calculate this change and evaluate the influence of increased TOC on the surface area of the frustum of a cone (tooth preparation). Figure 2c shows a superimposed image of Figure 2a,b, where α is the angulation between the long axis of the tooth and the axial wall. The “c” measurement shows the reduction from radius “b” needed to yield radius “a_1_.” The following formulas can be used to calculate radius “a_1_.”

$$\alpha \;=\;\;1/2\;\mathrm{TOC}c\;=\;\sin\left(\alpha \right)a_{1}\;=\;b\;-\;c.$$

### 2.1 Experiment

For documenting the student progress, ivory teeth prepared by students along with their faculty‐evaluated assessment form during preclinical fixed prosthodontics are kept at UB SDM. The preparations used in this study are selected from the previous fixed prosthodontics courses. Preparations were evaluated by calibrated faculty during practical exams and prior to the start of this study.

Eighty teeth (Kilgore International, Inc., Coldwater, MI, USA) prepared by dental students were selected for development of this assessment strategy. Preparations were selected based on faculty‐evaluated assessment forms in order to include a range of prepared teeth with “excellent,” “standard,” and “standard not met” scores for each criterion. Teeth were selected from preparations for complete cast crown of tooth no. 46, metal ceramic crown of teeth nos 24 and 46, and all ceramic crown of tooth no. 21, each 20. Prior to the fixed prosthodontics course, the course director prepared a tooth for student observation for each restoration following the same criteria. These preparations were defined as the standard preparations. The design and the amount of reduction for each tooth are presented in Table 1.

**Table 1:** Overview of the design for each tooth preparation

Preparation	Tooth	Amount of reduction
CCC	Mandibular first molar–tooth 46	Chamfer finish line width ≈ 0.5 mm, functional cusp ≈ 1.5 mm, nonfunctional cusp ≈ 1 mm
MCC	Maxillary first premolar–tooth 24	Lingual chamfer finish line width ≈ 0.5 mm, buccal shoulder finish line width ≈ 1.2 mm, functional and nonfunctional cusps ≈ 2 mm
MCC	Mandibular first molar–tooth 46	Lingual chamfer finish line width ≈ 0.5 mm, buccal chamfer finish line width ≈ 0.8 mm, functional cusp ≈ 2 mm, nonfunctional cusp ≈ 1 mm
ACC	Maxillary central incisor–tooth 9	Modified shoulder finish line width ≈ 1 mm, incisal edge reduction ≈ 1.5 mm, lingual reduction ≈ 1.5 mm

*Note*. CCC = complete cast crown; MCC = metal ceramic crown; ACC = all ceramic crown.

Student and standard preparations were digitally recorded using an intraoral scanner (Planmeca Corp., Helsinkli, Finland). The student preparations and standard preparations were uploaded into the Compare software (Planmeca/E4D Technologies). The margin, axial wall base, and occlusal table were defined, and the occlusal angle was set for the student preparations using the software. Comparison% was then calculated for student preparations versus standard preparations at a tolerance of 350 μm. Additionally, the average FLW, TOC, and AWH for each student preparation were calculated independent of the standard preparation using the software. For maxillary central incisors, the average AWH was not calculated; instead, mid‐lingual AWH was measured using a digital ruler tool available in the Compare software. Finally, the undercut presence or absence was evaluated for each preparation.

Table 2 aligns the criteria from faculty assessments and the criteria quantified by the Compare software. The values computed using the software were descriptively compared with faculty assessments and grouped for each criterion. Mathematically, FLL and the amount of O/IR were found to have the highest impact on the surface area. The sum of the FLL and the amount of O/IR criteria evaluated by the faculty was compared with the respective comparison% calculated using Compare software.

**Table 2:** Alignment of criteria from faculty and Compare software assessments

Faculty assessment	Compare software assessment
Occlusal/incisal reduction + finish line location	Comparison percentage
Finish line width	Finish line width average
Axial wall height	Axial wall height average (for anterior tooth, mid‐lingual axial wall height)

## 3 RESULTS

Video S1 depicts a Microsoft Excel file containing the formulas shown in Section 2. When the value of “h” increases or decreases, the amount of surface area changes considerably. Changes in “h” are influenced by the magnitude of the O/IR and the FLL. However, if radius “a” or “b” increases or decreases, the amount of surface area does not change significantly. Changes in radii “a” and “b” are influenced mostly by changes in TOC and FLW, respectively. The mathematical calculation demonstrates the minimal influence an increase in TOC has on the total surface area of the frustum of a cone.

**Figure 3:**
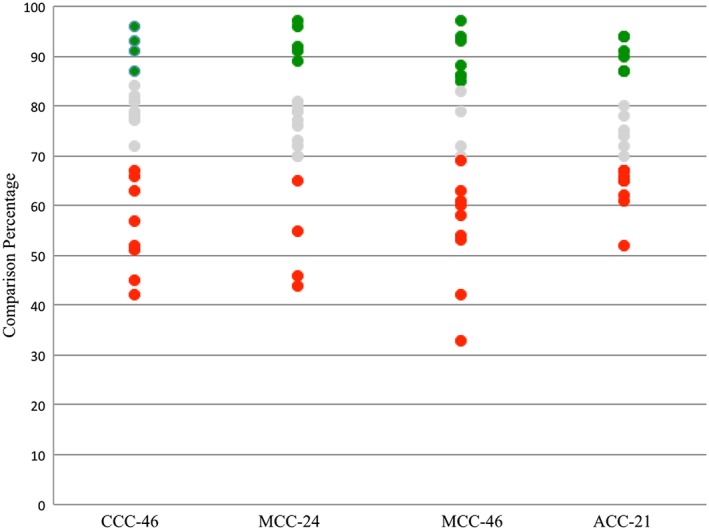
Comparison percentage for each tooth preparation for crowns calculated using Compare software for complete cast (CCC), metal ceramic (MCC), and all ceramic (ACC). Colors indicate the outcome of the faculty assessment. (Green: excellent; gray: standard; red: standard not met)

**Figure 4:**
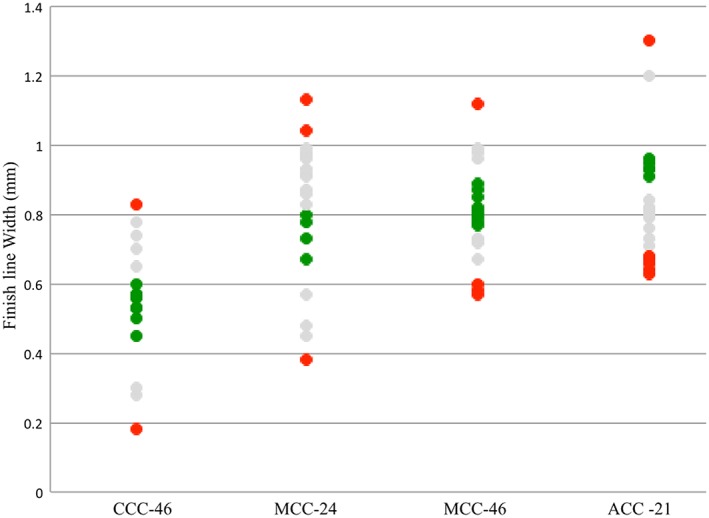
Average finish line width (mm) for each tooth preparation for crowns calculated using Compare software for complete cast (CCC), metal ceramic (MCC), and all ceramic (ACC). Colors indicate the outcome of the faculty assessment. (Green: excellent; gray: standard; red: standard not met)

**Figure 5:**
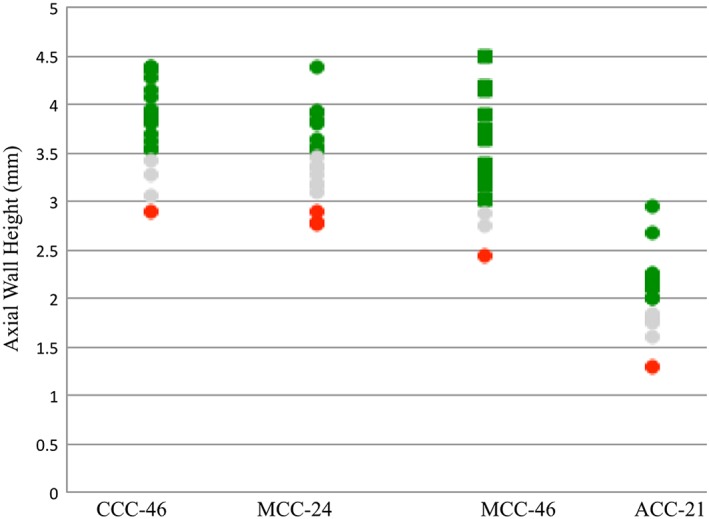
Average axial wall height (mm) for each tooth preparation for crowns calculated using Compare software for complete cast (CCC), metal ceramic (MCC), and mid‐lingual axial wall height (mm) for all ceramic (ACC). Colors indicate the outcome of the faculty assessment. (Green: excellent; gray: standard; red: standard not met)

**Figure 6:**
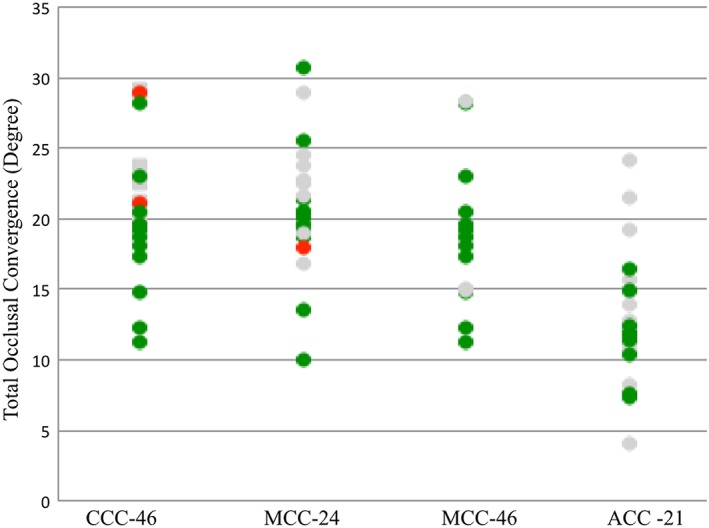
Average total occlusal convergence (°) for each tooth preparation for crowns calculated using Compare software for complete cast (CCC), metal ceramic (MCC), and all ceramic (ACC). Colors indicate the outcome of the faculty assessment. (Green: excellent; gray: standard; red: standard not met)

**Figure 7:**
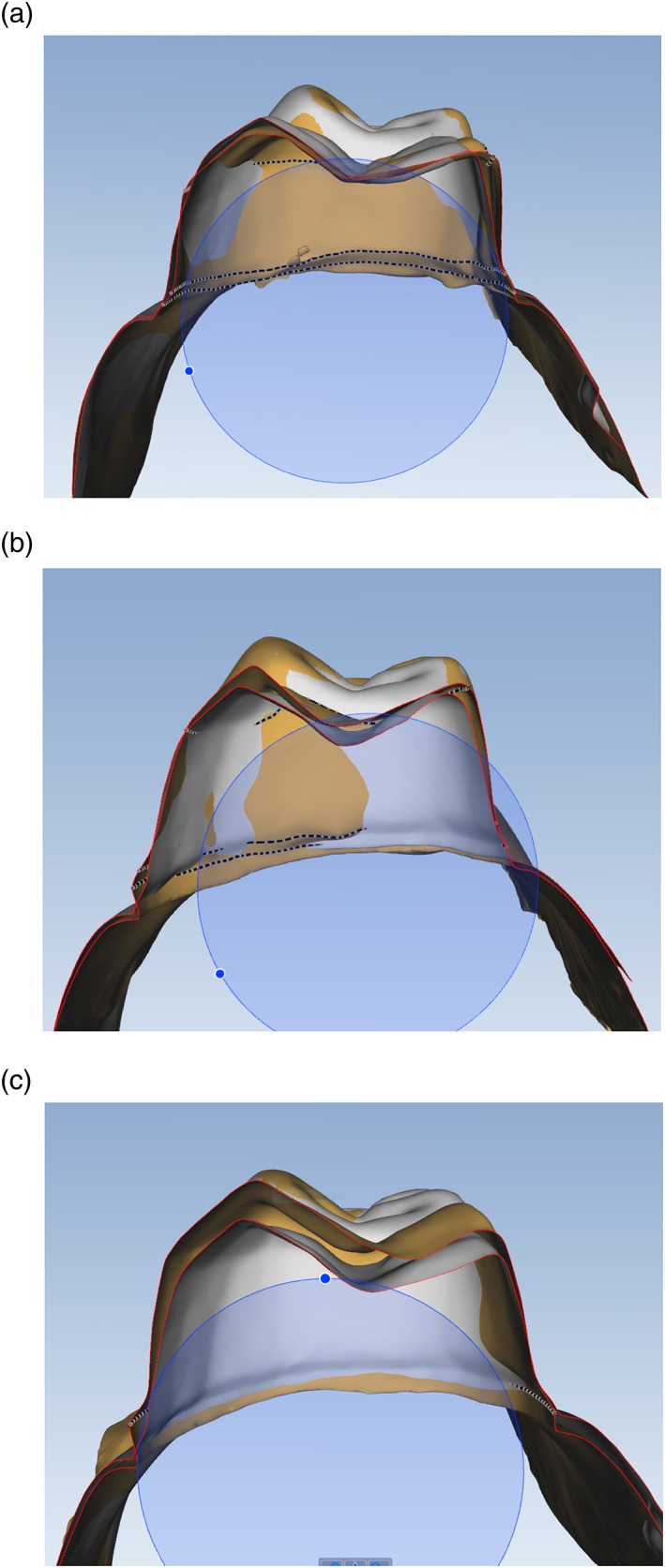
Comparison percentage of student complete cast crown preparations (beige color) superimposed on the standard complete cast crown preparation (gray color). (a) Student preparation with an “excellent” finish line location and occlusal reduction leading to a comparison percentage of 87%. (b) Student preparation with an “excellent” finish line location and a “standard” occlusal reduction leading to a comparison percentage of 83%. (c) Student preparation with an “excellent” finish line location and a “standard not met” occlusal reduction leading to a comparison percentage of 48%

Figures 3, 4, 5, 6 show data obtained from Compare software analyses. These data include the comparison%, average of FLW, AWH, and TOC values. The data are grouped based on their respective faculty assessment criteria. According to the mathematical calculations, the AWH has the greatest influence on the surface area of prepared teeth. As a result, the sum of FLL and the amount of O/IR assessed by faculty members was used to place tooth preparations into assessment categories (excellent, standard, or standard not met). Figure 3 shows the comparison% for each preparation categorized by these faculty assessments. In this study, preparations were considered excellent when faculty deemed both the amount of O/IR and FLL to be excellent (Figure 7a). Preparations were deemed standard when faculty assessed both the amount of O/IR and FLL to be standard or when one criterion was graded standard and the other excellent (Figure 7b). Preparations were scored as standard not met when one or both criteria were graded standard not met by the faculty (Figure 7c).

**Figure 8:**
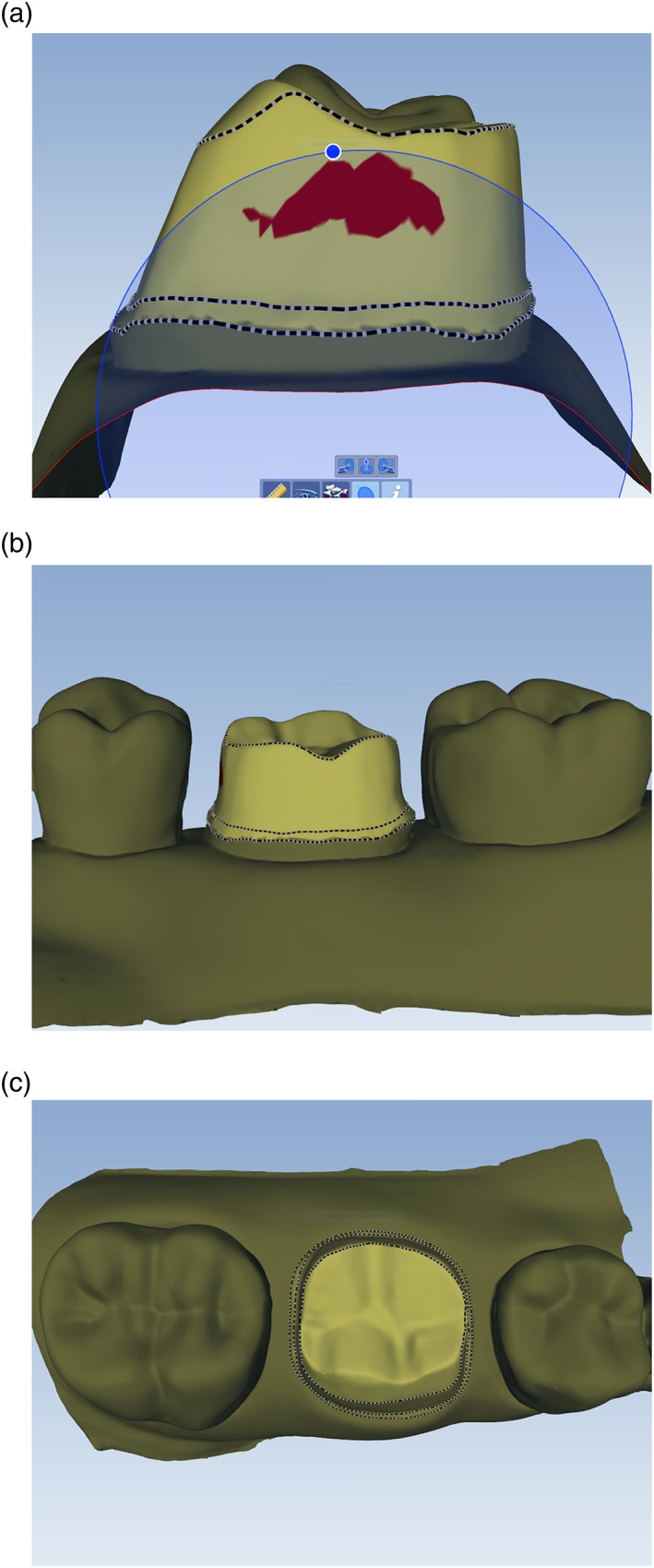
Complete cast crown preparation with absence of undercut. (a) Mesial view, compare software falsely detecting undercut on the mesial axial wall (red). (b) Lingual view. (c) Occlusal view

Figures 3, 4, 5, 6 categorize data obtained from the Compare software based on faculty assessment of FLW, AWH (or for tooth no. 21, mid‐lingual AWH), and TOC. Faculty assessment revealed that 5 of 80 student preparations exhibited undercut, whereas the Compare software identified undercut in 57 of 80 student preparations (Figure 8).

## 4 DISCUSSION

Simplified mathematical calculations describing tooth preparation demonstrate that for the same tooth, when the AWH “h” changes, the surface area of the preparation changes considerably. Video S1 shows that a 0.5‐mm increase in “h” has a substantial influence on the surface area of a prepared tooth. In dentistry, changes in AWH occur as a result of changes in the amount of O/IR and in FLL. In addition, when “h” is constant, the radius “a” for the same tooth preparation decreases to “a_1_” with an increase in TOC. Video S1 also shows that when “α” (angulation between the long axis of the tooth and the axial wall) increases from 3° to 10° and 20°, only minor changes occur in the total surface area.

Descriptive results from this study show that the comparison% can serve as a surrogate for the amount of O/IR and the FLL for student tooth preparations. Analysis of data from 80 prepared teeth revealed that comparison% ≥85% were associated with excellent faculty evaluations for the amount of O/IR and the FLL. However, 85 > comparison% ≥ 70 and comparison% < 70 were accounted for standard and standard not met in faculty evaluations, respectively.

Results for average FLW from Compare software were grouped by FLW from faculty assessment in Table 3. Table 4 shows the average FLW after preparation under ideal circumstances based on the diameter of the cutting instruments and the traditional assessment form used in the UB SDM preclinical fixed prosthodontics course. Regarding preparations with two finish line designs (preparations for metal ceramic crown), the averages shown in Table 4 are accurate if each finish line design is considered to cover 180° of the tooth. Results of this descriptive ranking for the average FLW (Table 3) align with ideal average FLL (Table 4).

**Table 3:** Suggested average finish line width (mm) range for each preparation

Preparation	N	S	E	S	N
CCC 46	X < 0.3	0.4 > X ≥ 0.3	0.6 ≥ X ≥ 0.4	0.8 ≥ X > 0.6	X > 0.8
MCC 24	X < 0.65	0.75 > X ≥ 0.65	0.95 ≥ X ≥ 0.75	1.1 ≥ X > 0.95	X > 1.1
MCC 46	X < 0.45	0.6 > X ≥ 0.45	0.8 ≥ X ≥ 0.6	1 ≥ X > 0.8	X > 1
ACC 21	X < 0.7	0.9 > X ≥ 0.7	1 ≥ X ≥ 0.9	1.2 ≥ X > 1	X > 1.2

*Note*. E = excellent; S = standard met; N = standard not met; CCC = complete cast crown; MCC = metal ceramic crown; ACC = all ceramic crown.

**Table 4:** Average ideal finish line width (mm) for each preparation based on the traditional assessment form

Preparation	Average finish line width (mm)
CCC 46	0.5
MCC 24	(0.5 + 1.2)/2 = 0.85
MCC 46	(0.5 + 0.8)/2 = 0.65
ACC 21	1

*Note*. CCC = complete cast crown; MCC = metal ceramic crown; ACC = all ceramic crown.

Our descriptive study did not address the quality of the finish line, a crucial factor in tooth preparation for complete coverage restorations. It is important for students to be attentive to the continuity and evenness of the finish line and the presence or absence of unsupported enamel, as well as to choose an appropriate finish line design for preparations with two finish line designs (as occurs in preparation for metal ceramic crowns). These qualitative criteria are subjective and cannot be easily measured or detected using available virtual evaluation software. Therefore, when using Compare software for assessment, in addition to average FLW, it is also important to implement a criterion evaluating quality of the finish line specific to the restoration.

Suggested average AWH for molars and premolars and suggested mid‐lingual AWH for anterior teeth are summarized in Table 5. Excellent preparations are suggested to be ≥3.5, ≥3, and ≥2 mm, for teeth nos 46, 24, and 21, respectively. The data obtained from the Compare software were grouped by faculty assessments of AWH. Goodacre et al. proposed 3 mm as the recommended AWH for premolars and anterior teeth prepared within the TOC range of 10°–20° (24). They recommended an AWH of 4 mm for molars because molars are usually prepared with greater convergence than anterior teeth, have a greater diameter than other teeth, and are located where occlusal forces are greater. AWH at the interproximal area of posterior teeth is less than that at the buccal and lingual areas because the cement‐enamel junction location is more occlusally located. In addition, the central groove area of a posterior tooth is located more cervically compared with the other parts of the occlusal table, resulting in short interproximal AWH. These anatomical features may have resulted in a lower than average AWH for molars in this study compared with the recommended AWH specified in the Goodacre, Campagni, and Aquilino (2001) study.

**Table 5:** Suggested average axial wall height (mm) for teeth nos 24 and 46 and suggested mid‐lingual axial wall height (mm) for tooth no. 21

Preparation	E	S	N
CCC/MCC 46	X ≥ 3.5	3.5 > X ≥ 3	X < 3
MCC 24	X ≥ 3	3 > X ≥ 2.5	X < 2.5
ACC 21	X ≥ 2	2 > X ≥ 1.5	X < 1.5

*Note*. E = excellent; S = standard met; N = standard not met; CCC = complete cast crown; MCC = metal ceramic crown; ACC = all ceramic crown.

**Figure 9:**
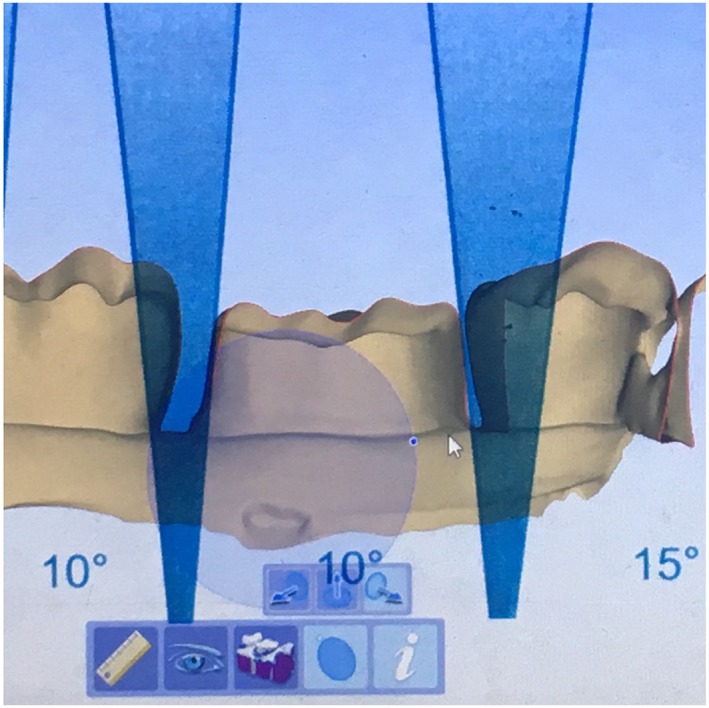
Loma Linda total occlusal convergence guide superimposed on a prepared premolar. The proposed method to measure total occlusal convergence of a tooth sliced mesio‐distally at the central groove

Average TOC results measured using Compare software in this study could not be clearly interpreted. Preparations with TOC assessed as excellent by faculty members had average TOC values from the Compare software that were less than 10° in some cases and more than 10° in others. For preparations in which TOC was assessed to be standard, the average TOC values from the Compare software were less than 10° in some cases or more than 10° in some cases and more than 20° in others. This wide variation might be because the software considers too many clinically irrelevant data points to calculate the average TOC. However, the authors suggest the use of the Compare software in evaluating the bucco‐lingual and mesi‐distal TOC in a manner different than that used here. Compare software has a “slice plane” tool that can be used to slice a three‐dimensional image of the preparation bucco‐lingually and mesio‐distally. A magnified clear Loma Linda TOC guide could be superimposed on the sliced plane in order to measure TOC at the selected slice (Figure 9). Pairing the Loma Linda TOC guide with Compare software could provide a less subjective means of TOC assessment.

The Compare software also failed to recognize the presence of clinically relevant undercut in the student preparations. These data suggest that the software measures detailed information in detecting undercut, but this detailed information does not appear to be clinically relevant.

Following this study and after the curriculum committee approval, syllabus was modified to incorporate the use of Compare software at UB SDM preclinical curriculum fixed prosthodontics. In this course, Tables 3, 4, and 5 were used to evaluate the amount of O/IR and FLL, average of FLW, and average of AWH, respectively. In addition, the faculty members assessed TOC, finish of the preparation, quality of the finish line, and adjacent teeth.

## 5 CONCLUSIONS

Compare software may be used to evaluate a complete coverage preparation by objective measurement. Within the limitations of this descriptive study, the following conclusions can be drawn:

1. Compare‐generated comparison% in 350‐μm tolerance can be used as quantitative measurement of the sum of the amount of O/IR and FLL.
2. Average FLW calculated using Compare software could serve as a quantitative measurement of FLW using the suggested ranges.
3. Ranges shown for the average AWH of posterior teeth and mid‐lingual AWH of anterior teeth can be used as criteria for quantification of AWH.
4. Average TOC and measurements of the presence or absence of undercut by Compare software should not be used to assess student preparations.

## CONFLICT OF INTEREST

None declared.

## Supporting information

Movie S1. Supporting information
